# Building a Global Evidence Base to Guide Policy and Implementation for Group Antenatal Care in Low- and Middle-Income Countries: Key Principles and Research Framework Recommendations from the Global Group Antenatal Care Collaborative

**DOI:** 10.1111/jmwh.13143

**Published:** 2020-10-03

**Authors:** Lindsay Grenier, Jody R. Lori, Blair G. Darney, Lisa Miyako Noguchi, Sheela Maru, Carrie Klima, Tiffany Lundeen, Dilys Walker, Crystal L. Patil, Stephanie Suhowatsky, Sabine Musange

**Affiliations:** 1 Maternal and Newborn Health, Jhpiego, Baltimore, Maryland; 2 Department of Health Behavior and Biological Science, School of Nursing, University of Michigan, Ann Arbor, Michigan; 3 Department of Obstetrics & Gynecology, Oregon Health & Science University, Portland, Oregon; 4 National Institute of Public Health, Center for Population Health Research, Cuernavaca, Mexico; 5 Department of Health Systems Design and Global Health, Icahn School of Medicine at Mount Sinai, New York, New York; 6 Department of Women, Children, and Family Health Sciences, College of Nursing, University of Illinois at Chicago, Chicago, Illinois; 7 Institute for Global Health Sciences, University of California San Francisco, San Francisco, California; 8 Department of Obstetrics, Gynecology, and Reproductive Health Sciences and Institute for Global Health Sciences, University of California San Francisco, San Francisco, California; 9 University of Rwanda, School of Public Health, Kigali, Rwanda

**Keywords:** Centering Pregnancy/group care, global health/international, antepartum care, public health, quality improvement

## Abstract

Evidence from high-income countries suggests that group antenatal care, an alternative service delivery model, may be an effective strategy for improving both the provision and experience of care. Until recently, published research about group antenatal care did not represent findings from low- and middle-income countries, which have health priorities, system challenges, and opportunities that are different than those in high-income countries. Because high-quality evidence is limited, the World Health Organization recommends group antenatal care be implemented only in the context of rigorous research. In 2016 the Global Group Antenatal Care Collaborative was formed as a platform for group antenatal care researchers working in low- and middle-income countries to share experiences and shape future research to accelerate development of a robust global evidence base reflecting implementation and outcomes specific to low- and middle-income countries. This article presents a brief history of the Collaborative’s work to date, proposes a common definition and key principles for group antenatal care, and recommends an evaluation and reporting framework for group antenatal care research.

## INTRODUCTION

In 2017 the global maternal mortality ratio was 211 maternal deaths per 100,000 live births, with 94% of these deaths taking place in low- and lower-middle-income countries.^1^ The United Nations Sustainable Development Goal 3.1 aims to reduce the global maternal mortality ratio to less than 70 per 100,000 live births with no country above 140 per 100,000 live births by 2030.^2^ To meet this goal more progress will need to be made in improving women’s health before and during pregnancy. Historically, global efforts to reduce maternal and neonatal mortality have focused on the first 24 hours after birth, when more than 40% of maternal and neonatal deaths have occurred.^3^ However, large-scale demographic, socioeconomic, and epidemiologic transitions in low- and middle-income countries are shifting the proportional contributions of direct (eg, hemorrhage, sepsis, eclampsia) and indirect causes of maternal mortality.^4,5^ Indirect causes include pre-existing conditions (eg, anemia, diabetes, or hypertension) or diseases that arise during pregnancy (eg, malaria) that are aggravated but not caused by pregnancy. Whereas direct obstetric causes of death often arise and are addressed at the time of birth, indirect causes often need to be addressed and managed during antenatal care. When optimally implemented, antenatal care provides woman-specific health promotion, disease prevention, screening, and management of complications.^6,7^ Adequate antenatal care is associated with decreased neonatal mortality.^8,9^ An analysis of 57 low- and middle-income countries found a 55% lower risk of neonatal mortality (hazard ratio, 0.45; 95% CI, 0.42–0.48) among women who attended at least 4 antenatal visits, including one in the first trimester.^8^

Unfortunately, women in low- and middle-income countries continue to receive antenatal care of inadequate quality with low coverage of essential interventions.^10–12^ Low-quality care, in turn, is associated with reduced antenatal care attendance,^13,14^ further reducing the odds that women will receive the care and information essential for reducing the risk of morbidity and mortality. Globally only 65% of pregnant women attend at least 4 antenatal care visits.^15^

In 2016, the World Health Organization (WHO) released the *WHO Recommendations on Antenatal Care for a Positive Pregnancy Experience*.^6^ The recommendations are framed around women’s experience of care, recognizing that the experience is an important driver of care-seeking behavior. WHO acknowledges that how antenatal care is delivered is as important as its content and recommends group antenatal care as a health system intervention to improve the utilization and quality of antenatal care “in the context of rigorous research.”^6(p 91)^ The qualified nature of this endorsement speaks to the quality of evidence regarding the effectiveness of group antenatal care that was available at the time the WHO recommendations were formed.

A single Cochrane review published in 2015 met WHO criteria to be considered as evidence for the effects of group antenatal care compared with individual antenatal care.^16^ The review offers some indication that group antenatal care may reduce the incidence of preterm birth in high-income countries (risk ratio, 0.75; 95% CI, 0.57–1.00; 3 trials, n = 1888) and lead to higher satisfaction among women (mean difference, 4.9; 95% CI, 3.1–6.7; 1 trial, n = 993). The Cochrane review includes only 4 studies involving 2350 women and notes inconsistent outcomes across trials. Furthermore, the evidence for both preterm birth and satisfaction are considered of low certainty by WHO grading standards.^6^ Other studies conducted in the United States have found positive associations between group antenatal care and increased antenatal care attendance, improved breastfeeding practices, and uptake of postpartum contraception.^17,18^

## THE GLOBAL GROUP ANTENATAL CARE COLLABORATIVE

Prior to release of the 2016 WHO antenatal care recommendations, various efforts were already underway to adapt and introduce group antenatal care models to address the needs of women and antenatal care providers in low- and middle-income countries. In October 2015, at the Global Maternal and Newborn Health Conference in Mexico City, Jhpiego organized a panel of group antenatal care researchers working in low- and middle-income countries. The individuals on the panel represented group antenatal care initiatives in low-income countries that were conducted by the University of Michigan (Ghana),^19–21^ the University of Illinois Chicago (Tanzania and Malawi),^22,23^ and the non-profit organization Possible (Nepal).^24^ Panel organizers, participants, and others embarking on group antenatal care research in low- and middle-income countries, including Jhpiego (Kenya and Nigeria),^25,26^ the University of California, San Francisco (Rwanda),^27–30^ and Instituto Nacional de Salud Pública, Mexico,^31^ met to exchange ideas and experiences. This group identified common challenges for research about group antenatal care in low- and middle-income countries (Table 1) and the need for an ongoing formalized mechanism to better coordinate among group antenatal care research projects in low- and middle-income country contexts.

In March 2016, the Global Group Antenatal Care Collaborative was formally created as a platform for researchers working in low- and middle-income countries to (1) share experiences, learning, and data collection tools (2) build consensus around defining and evaluating group antenatal care, and (3) advocate for more group antenatal care research in low- and middle-income countries. Prior to the Collaborative’s formation little was known about group antenatal care implementation, sustainability, or performance in low- and middle-income country contexts. The founding members saw an opportunity to accelerate learning and innovation around group antenatal care in low- and middle-income countries by working together to codify its essential elements, research tools, and priorities.

### Global Group Antenatal Care Collaborative Consensus Statement for Coordinated Research to Accelerate Learning

The Global Group Antenatal Care Collaborative believes that group antenatal care has transformational potential. However, that potential will only be realized if coordinated research can clarify the relationships between model and implementation characteristics, context, and outcomes. Since its inception the Collaborative has worked toward mitigating the challenges outlined in Table 1 with the mission to accelerate and consolidate learning to identify scalable models and implementation strategies that produce measurable improvements in antenatal care quality and experience. At the inaugural meeting of the Collaborative, key principles for group antenatal care were defined, and an evaluation and reporting framework was articulated. In this article, members of the Collaborative share the definitions and framework to disseminate these ideas and call for participation among interested readers. The Collaborative previously published the key principles of group antenatal care on its public website. In this article, the Collaborative shares its research framework for the first time.

### A Common Definition with Guiding Principles for Group Antenatal Care in Low- and Middle-Income Countries

The Global Group Antenatal Care Collaborative recognizes that group antenatal care models in low- and middle-income countries need to be customized to local contexts and local priorities to ensure ownership, sustainability, and scalability. Furthermore, research has not yet established ideal parameters for some aspects of implementation (eg, optimal meeting dose and group composition), which may also vary by context and program priorities. However, the Collaborative believes a common definition of group antenatal care in low- and middle-income countries is needed to create a credible and useful body of evidence. A definition that includes a minimum set of defining characteristics and key principles based on Collaborative members’ expertise in group antenatal care, other successful group interventions, and theories of social and behavioral change is proposed and described in Table 2.

### Evaluation and Reporting Framework for Group Antenatal Care in Low- and Middle-Income Countries

Because group antenatal care is unlikely to look the same across settings, a unified evaluation and reporting framework for group antenatal care can help ensure that research priorities align with the data needed by policy and funding decision makers and that adequate information is reported to consider the relative impact of context, implementation strength, and specific model characteristics on outcomes. The Collaborative urges use of the framework, presented in Table 3, which was developed to be responsive to the needs and interests of local health systems and ministries of health, global normative agencies (ie, WHO), and international donors.

#### Description of Model and Framework and Programmatic Elements

Group antenatal care models and frameworks will vary by setting both by necessity and because of innovation. The Collaborative urges researchers to explicitly describe key components of each model such as the number, timing, and structure of meetings and group composition. In addition, researchers should comment on how each key principle of group antenatal care (Table 2) has or has not been addressed within their model. Careful reporting of programmatic elements such as the type of training and ongoing support offered to facilities initiating group antenatal care is also recommended. Clear reporting will allow for a more nuanced understanding of the relationships between outcomes, context, and model specifics. This enhanced understanding will also help to better adapt and refine the intervention by setting and efficiently scale successful models within appropriate contexts.

#### Client-Focused Outcomes

The Collaborative has not, as yet, endorsed specific indicators for client-focused outcomes. Instead, 6 priority subdomains of outcomes listed in Table 3 are highlighted. Researchers are encouraged to use previously validated research tools and standard indicators where available. Recognizing that most studies will not be powered to detect significant differences in rare outcomes, it is recommended that researchers collect and report significant maternal and neonatal outcomes such as mortality so that data may be combined in future meta-analyses. In all cases, analyses of client outcomes should aim to disentangle confounders, mediators, and effect modifiers of this service delivery model.

#### Health System Considerations

When a health system considers widespread adoption of research findings into practice, potential changes in outcomes must be balanced with potential changes in the health system itself. Human resources allocation is of particular interest in low- and middle-income country contexts in which health care staff shortages are common. If group antenatal care is associated with improved outcomes, decision makers will need additional information on necessary health system inputs and impacts to develop appropriate policies and guidelines and dedicate sufficient resources. This is particularly important as many low- and middle-income countries have decentralized health systems, and subnational health officials ultimately will need guidance from their respective ministries of health to implement group antenatal care where feasible, plan activities in annual workplans, and budget adequate resources. Gathering and reporting this information for all group antenatal care projects will accelerate understanding of these important issues and subsequently speed or prevent scale-up as appropriate.

## INVITATION TO LOW- AND MIDDLE-INCOME COUNTRIES GROUP ANTENATAL CARE RESEARCHERS AND IMPLEMENTERS

Current Global Group Antenatal Care Collaborative members are committed to using the evaluation framework outlined in this article to advance our shared research agenda. Members of the Collaborative actively share theoretical frameworks, experiences, materials, evaluation frameworks, and data collection tools, aligning data collection where possible to strengthen the external validity of findings. Collaborative members have contributed significantly to the global evidence base for group antenatal care in low- and middle-income countries since formation of the Collaborative, confirming the feasibility and acceptability of group antenatal care in multiple low- and middle-income countries and finding associations with increased quality of care, facility-based delivery, antenatal care attendance, uptake of postpartum family planning, health literacy, and pregnancy-related empowerment.^20–24,29,30^

## CONCLUSION

In low- and middle-income countries, group antenatal care has the potential to transform the dominant antenatal care service delivery model and provide a better care experience for women and antenatal care providers. The Global Group Antenatal Care Collaborative has a definition, key principles, and research framework for implementors and investigators presented in this article. All groups and individuals engaged in group antenatal care research in low- and middle-income countries are invited to join this collaboration to expedite the development of a robust evidence base on group antenatal care research in low- and middle-income countries. More information about the Global Group Antenatal Care Collaborative is available at its website (ganccollaborative.com). The Collaborative hopes that ultimately its work results in an increase in high-quality evidence regarding the effectiveness of group antenatal care across the globe that ultimately contributes to greater antenatal care access, quality, and coverage for all pregnant women.

## Figures and Tables

**Table 1.** Research Challenges Related to Group Antenatal Care in Low- and Middle-Income Countries

Challenge	Background
Published research not reflective of priorities in low- and middle-income countries	The published group antenatal care evidence base all but exclusively represented high-income country settings. High- income countries have different disease burdens, health system resources, and health priorities compared with low- and middle-income countries. Group antenatal care research from high-income countries lacked data related to common low- and middle-income countries’ priorities such as facility-based delivery and use of malaria prophylaxis.
Published implementation research not reflective of low- and middle-income countries’ constraints and opportunities	High-income countries and low- and middle-income countries often have different challenges and opportunities related to health care. For example, group antenatal care results from high-income countries have been based on implementation models impractical for low- and middle-income countries where literacy rates may be low and women generally attend far fewer antenatal care visits. Likewise, infrastructure, staffing, antenatal care provider scopes of work, and financing differ substantially by setting.
No commonly agreed-upon research priorities or data collection tools	There were no norm setting or donor agencies advocating for a standardized approach to group antenatal care research. Understanding the potential and limits of group antenatal care in low- and middle-income countries could be accelerated if multiple trials and projects collected similar information in similar ways, enhancing the ability to meta-analyze data as well as compare and contrast settings and implementation strategies.
No commonly agreed-upon definition of group antenatal care, creating potential for confusion and confounding with other interventions	There was no explicit definition of group antenatal care in use by those adapting the intervention for low- and middle-income countries. Group antenatal care was being confused with both “Care Groups” (a community-based intervention with similar educational and peer support elements, but no clinical care) and “Group Health Talks” (a common practice providing didactic health promotion lectures in antenatal waiting areas).

**Table 2.** Global Group Antenatal Care Collaborative Definition of Group Antenatal Care in Low- and Middle-Income Countries^a^

Group Antenatal Care Elements	Group Antenatal Care Key Principles
Clinical assessment and care provided for all routine antenatal care services	Plan for stability of group members and facilitators
	Have a plan and purpose for each session while remaining responsive to group interests
Participatory, facilitated learning Peer support	Capitalize on group processes that use nonhierarchical, client-centered, participatory methods
	Provide the widest range of care possible within the group setting
	Promote empowerment, self-efficacy, reflection, and planned action through specific activities (eg, clinical self-assessment and activities designed to improve health literacy)
	Promote peer-to-peer learning, support, group identity, and cohesion

a After the first (individual) antenatal care visit, some of or all subsequent antenatal care visits are replaced by a series of group visits (ie, meetings) for pregnant women and at least one trained facilitator. Each visit or meeting includes all 3 elements and follows the key principles.

**Table 3.** Recommended Research and Reporting Framework for Group Antenatal Care Research in Low- and Middle-Income Countries

Domain	Subdomain	Illustrative Components
Description of model and framework and programmatic elements	Participants	Number of women per cohort; common characteristics of cohort (eg, gestational age or HIV status); number, cadre, and training of facilitators
	Dose and schedule	Length and frequency of group ANC meetings, total number of planned ANC contacts (individual and group)
	Meeting content and methodology	Topics covered, common components of meetings, if and how the model addresses key principles outlined in Table 2
	Implementation plan	Training, mentoring, quality improvement tools or activities
Client-focused outcomes	Health service utilization	ANC and postnatal care attendance, facility-based delivery, family planning uptake
	Quality of care: provision	Screening: blood pressure, hemoglobin, urine dipsticks, HIV and syphilis testing
		Prevention: intermittent preventive treatment (of malaria) in pregnancy, tetanus toxoid
	Quality of care: experience (antenatal care providers and women)	Satisfaction, respectful care
	Health literacy and self-efficacy	Ability to name danger signs, confidence in own ability to act on danger signs
	Uptake of healthy behaviors	Use of iron-folic acid supplements and long-lasting insecticide treated mosquito net, immediate and exclusive breastfeeding, optimal birth spacing
	Key context-specific maternal and neonatal outcomes	Stillbirth, preterm birth, low birth weight, maternal and neonatal mortality, maternal anemia at time of birth, malaria in pregnancy
Health system considerations	Service delivery impacts	Staffing requirements, proportion of ANC clients receiving group ANC, wait times and availability for non-ANC services
	Scalability and sustainability	Costing, training and supervision requirements, infrastructure needs
	Policy implications	ANC guideline changes, financing mechanisms

Abbreviation: ANC, antenatal care.

## Quick Points

- The World Health Organization currently recommends group antenatal care as a health system intervention to improve the utilization and quality of antenatal care only in the context of rigorous research.
- A robust group antenatal care evidence base is lacking for the unique needs of low- and middle-income countries.
- The Global Group Antenatal Care Collaborative was formed in 2016 to facilitate research and learning on group antenatal care to improve the delivery of and outcomes associated with antenatal care at scale in low- and middle-income countries.
- The Collaborative encourages use of the group antenatal care evaluation and reporting framework presented here to improve the global evidence base of group antenatal care in low- and middle-income countries.

## References

[1] World Health Organization. Trends in Maternal Mortality: 2000 to 2017. Estimates by WHO, UNICEF, UNFPA, World Bank Group and the United Nations Population Division. Geneva, Switzerland: World Health Organization; 2019.

[2] Goal 3: Ensure healthy lives and promote well-being for all at all ages. United Nations Sustainable Development Goals website. Accessed December 10, 2019. un.org/sustainabledevelopment/health/

[3] Lawn JE, Blencowe H, Oza S, Every newborn: progress, priorities, and potential beyond. Lancet. 2014;384(9938):189–205. 24853593 10.1016/S0140-6736(14)60496-7

[4] Souza JP, Gülmezoglu AM, Vogel J, Moving beyond essential interventions for reduction of maternal mortality (the WHO Multicountry Survey on Maternal and Newborn Health): a cross-sectional study. Lancet. 2013;381(9879):1747–1755. 23683641 10.1016/S0140-6736(13)60686-8

[5] Graham W, Woodd S, Byass P, Diversity and divergence: the dynamic burden of poor maternal health. Lancet. 2016;388(10056):2164–2175. 27642022 10.1016/S0140-6736(16)31533-1

[6] World Health Organization. WHO Recommendations on Antenatal Care for a Positive Pregnancy Experience. Geneva, Switzerland: World Health Organization; 2016. 28079998

[7] De Masi S, Bucagu M, Tuncalp Ö, Integrated person-centered health care for all women during pregnancy: implementing World Health Organization recommendations on antenatal care for a positive pregnancy experience. Glob Health Sci Pract. 2017;5(2):197–201. 28655799 10.9745/GHSP-D-17-00141 PMC5487083

[8] Doku DT, Neupane S. Survival analysis of the association between antenatal care attendance and neonatal mortality in 57 low- and middle-income countries. Int J Epidemiol. 2017;46(5):1668–1677. 29040531 10.1093/ije/dyx125 PMC5837573

[9] Kuhnt J, Vollmer S. Antenatal care services and its implications for vital and health outcomes of children: evidence from 193 surveys in 69 low-income and middle-income countries. BMJ Open. 2017;7(11):e017122. 10.1136/bmjopen-2017-017122 PMC5695442 29146636

[10] Hodgins S, D’Agostino A. The quality-coverage gap in antenatal care: toward better measurement of effective coverage. Glob Health Sci Pract. 2014;2(2):173–181. 25276575 10.9745/GHSP-D-13-00176 PMC4168625

[11] Benova L, Tunçalp Ö, Moran AC, Campbell OMR. Not just a number: examining coverage and content of antenatal care in low-income and middle-income countries. BMJ Glob Health. 2018;3(2):e000779. 10.1136/bmjgh-2018-000779 PMC5898334 29662698

[12] Kanyangarara M, Munos MK, Walker N. Quality of antenatal care service provision in health facilities across sub-Saharan Africa: evidence from nationally representative health facility assessments. J Glob Health. 2017;7(2):e021101. 10.7189/jogh.07.021101 PMC5680531 29163936

[13] Simkhada B, van Teijlingen ER, Porter M, Simkhada P. Factors affecting the utilization of antenatal care in developing countries: systematic review of the literature. J Adv Nurs. 2008;61(3):244–260. 18197860 10.1111/j.1365-2648.2007.04532.x

[14] Downe S, Finlayson K, Tunçalp Ö, Gülmezoglu AM. Provision and uptake of routine antenatal services: a qualitative evidence synthesis. Cochrane Database Syst Rev. 2019;(6):CD012392. 31194903 10.1002/14651858.CD012392.pub2 PMC6564082

[15] Antenatal care. UNICEF website. Accessed December 10, 2019. https://data.unicef.org/topic/maternal-health/antenatal-care/

[16] Catling CJ, Medley N, Foureur M, Group versus conventional antenatal care for women. Cochrane Database Syst Rev. 2015;(2):CD007622. 25922865 10.1002/14651858.CD007622.pub3 PMC6465187

[17] Hale N, Picklesimer AH, Billings DL, Covington-Kolb S. The impact of Centering Pregnancy group prenatal care on postpartum family planning. Am J Obstet Gynecol. 2014;210(1):50.e1–50.e7. 24018309 10.1016/j.ajog.2013.09.001

[18] Trotman G, Chhatre G, Darolia R, Tefera E, Damle L, Gomez-Lobo V. The effect of Centering Pregnancy versus traditional prenatal care models on improved adolescent health behaviors in the perinatal period. J Pediatr Adolesc Gynecol. 2015;28(5):395–401. 26233287 10.1016/j.jpag.2014.12.003

[19] Kabue MM, Grenier L, Suhowatshky S, Group versus individual antenatal and first year postpartum care: study protocol for a multicountry cluster randomized controlled trial in Kenya and Nigeria. Gates Open Res. 2019;2:56. 30706056 10.12688/gatesopenres.12867.1 PMC6350506

[20] Grenier L, Suhowatsky S, Kabue MM, Impact of group antenatal care (G-ANC) versus individual antenatal care (ANC) on quality of care, ANC attendance and facility-based delivery: a pragmatic cluster-randomized controlled trial in Kenya and Nigeria. PLoS One. 2019;14(10):e0222177. 31577797 10.1371/journal.pone.0222177 PMC6774470

[21] Lori JR, Chuey M, Muro-Kramer ML, Ofosu-Darkwah H, Adanu RMK. Increasing postpartum family planning uptake through group antenatal care: a longitudinal prospective cohort design. Reprod Health. 2018;15(1):208. 30558677 10.1186/s12978-018-0644-y PMC6296041

[22] Lori JR, Ofosu-Darkwah H, Boyd CJ, Banerjee T, Adanu RMK. Improving health literacy through group antenatal care: a prospective cohort study. BMC Pregnancy Childbirth. 2017;17(1):228. 28705179 10.1186/s12884-017-1414-5 PMC5513199

[23] Lori JR, Munro ML, Chuey MR. Use of a facilitated discussion model for antenatal care to improve communication. Int J Nurs Stud. 2016;54:84–94. 25862409 10.1016/j.ijnurstu.2015.03.018 PMC4592694

[24] Patil CL, Abrams ET, Klima C, CenteringPregnancy-Africa: a pilot of group antenatal care to address millennium development goals. Midwifery. 2013;29(10):1190–1198. 23871278 10.1016/j.midw.2013.05.008 PMC3786019

[25] Patil CL, Klima CS, Steffen AD, Leshabari SC, Pauls H, Norr KF. Implementation challenges and outcomes of a randomized controlled pilot study of a group prenatal care model in Malawi and Tanzania. Int J Gynecol Obstet. 2017;139(3):290–296. 10.1002/ijgo.12324 PMC5673548 28905377

[26] Thapa P, Bangura AH, Nirola I, The power of peers: an effectiveness evaluation of a cluster-controlled trial of group antenatal care in rural Nepal. Reprod Health. 2019;16(1):150. 31640770 10.1186/s12978-019-0820-8 PMC6805428

[27] Sayinzoga F, Lundeen T, Gakwerere M, Use of a facilitated group process to design and implement a group antenatal and postnatal care program in Rwanda. J Midwifery Womens Health. 2018;63(5):593–601. 10.1111/jmwh.12871 PMC6220997 30251304

[28] Musange SF, Butrick E, Lundeen T. Group antenatal care versus standard antenatal care and effect on mean gestational age at birth in Rwanda: protocol for a cluster randomized controlled trial. Gates Open Res. 2019;3:1548. 31656954 10.12688/gatesopenres.13053.1 PMC6792348

[29] Lundeen T, Musange S, Azman H, Nurses’ and midwives’ experiences of providing group antenatal and postnatal care at 18 health centers in Rwanda: a mixed methods study. PLoS One. 2019;14(7):e0219471. 31295335 10.1371/journal.pone.0219471 PMC6622527

[30] Musabyimana A, Lundeen T, Butrick E, Before and after implementation of group antenatal care in Rwanda: a qualitative study of women’s experiences. Reprod Health. 2019;16(1):90. 31248425 10.1186/s12978-019-0750-5 PMC6595554

[31] Heredia-Pi IB, Fuentes-Rivera E, Andrade-Romo Z, The Mexican experience adapting CenteringPregnancy: lessons learned in a publicly funded health care system serving vulnerable women. J Midwifery Womens Health. 2018;63(5):602–610. 10.1111/jmwh.12891 PMC6220951 30199143

